# Exploring the Time to Onset and Early Predictors of Poststroke Spasticity Combined With Surface Electromyography: Protocol for a Nested Case-Control Study

**DOI:** 10.2196/65829

**Published:** 2025-08-05

**Authors:** Simeng Song, Shiliang Wang, Shanshan Zeng, Wenqing Wu, Lingying Wu, Xukun Tang, Xiongxing Sun, Dahua Wu, Le Xie

**Affiliations:** 1 Graduate School Hunan University of Traditional Chinese Medicine Changsha China; 2 Hunan Provincial Hospital of Integrated Traditional Chinese and Western Medicine (Hunan Academy of Chinese Medicine Affiliated Hospital) Changsha China

**Keywords:** nested case-control study, poststroke spasticity, stroke, surface electromyography, early predictor

## Abstract

**Background:**

Poststroke spasticity (PSS) is a frequent sequela in patients who have experienced stroke. This form of paralysis is more prevalent compared to other poststroke sequelae and is among the most challenging and complex symptoms to manage. Surface electromyography (sEMG) can reflect the physiological information of muscles in real time and is highly beneficial in diagnosing neuromuscular diseases in clinical medicine.

**Objective:**

This study aimed to investigate the timing of poststroke limb spasms using a nested case-control study combined with sEMG and to identify and predict factors of PSS at an early stage.

**Methods:**

This was a nested case-control study. Participants were assessed within 24 hours of the onset of hospitalization using a standardized case report form to evaluate general patient information and clinical data related to cerebral infarction and imaging. Upon inclusion, patients were assessed after 1, 2, 4, 8, and 12 weeks, using the Modified Ashworth Scale (MAS) for spasticity severity, root mean square values from sEMG for limb spasm severity, and the simplified Fugl-Meyer (S-FM) Assessment for limb motor function. Patients who experienced spasticity within 12 weeks were assigned to the spasticity group, whereas those who did not experience spasticity were assigned to the control group. Unmatched case grouping was implemented. Data with normal distribution were analyzed using the *t* test, while data with nonnormal distribution were analyzed using the rank-sum test; categorical data were analyzed using the chi-square test, rank-sum test, or Fisher exact test. Logistic regression analysis was used to investigate factors affecting treatment outcomes. Data processing, analysis, and visualization were conducted using Statistical Package for the Social Sciences software (version 26.0; IBM Corp).

**Results:**

This study is funded by the Chinese Association of Ethnic Medicine and began participant recruitment and registration in November 2023. The study is currently ongoing, with 66 participants enrolled as of March 2025.

**Conclusions:**

This study selected a diagnostic method combining sEMG and subjective scales to determine PSS, aiming to eliminate diagnostic errors caused by subjective assessments. This study adopted a nested case-control study method, which has minimal information bias, allowing for the inference of causal relationships between exposure and disease.

**Trial Registration:**

Chinese Clinical Trial Registry ChiCTR2300077121; https://www.chictr.org.cn/showproj.html?proj=205037

**International Registered Report Identifier (IRRID):**

DERR1-10.2196/65829

## Introduction

Stroke is currently the leading cause of disability and death in China, with a high incidence, high disability rate, high mortality rate, and high recurrence rate [1]. In China, approximately 3 million new patients with stroke are diagnosed annually, and 70%-80% of stroke survivors experience residual limb paralysis, which prevents them from living independently. Poststroke spasticity (PSS) is the most prevalent of these disabilities [2]. PSS is the result of stroke-induced damage to upper motor neurons, causing the loss of basic voluntary motor control by the advanced motor centers of the cerebral cortex and the inhibition of the primitive functions of the lower motor centers, resulting in a spastic state [3]. Muscle tone is the force generated by the interaction of muscle cells, typically measured by the resistance experienced during passive muscle stretching. However, these methods are unable to quantify muscle tone objectively [4].

Related researches suggest that surface electromyography (sEMG) can reflect muscle tone mechanisms through the threshold of muscle reflex electromyography, enabling direct, objective, timely, and accurate descriptions of spastic muscle tone [5]. Many studies also demonstrate that sEMG is crucial for investigating muscle contraction status and evaluating the degree of spasticity [4,6,7]. It quantitatively detects and evaluates the synchronization and contraction levels of different muscle groups involved in activities, thereby enhancing its utility in disease in the diagnosis and evaluation of diseases [8].

It is estimated that approximately 25% of patients experience spasticity within 2 weeks after cerebral infarction based on existing observational data. A total of 9 recent studies were reviewed and summarized by Sunnerhagen et al [9], which evaluated the spasticity status at different stages after stroke. The research findings emphasize the necessity of conducting follow-ups with patients at an increased risk of severe spasticity to facilitate the initiation of appropriate spasticity treatment and prevent adverse outcomes. Therefore, this clinical study aimed to improve the prognosis of patients with PSS by exploring the time of PSS occurrence through a nested case-control (NCC) study, early identification, and prediction of PSS factors. Our research plan and informed consent form were submitted to the Ethics Committee of the Affiliated Hospital of Hunan University of Chinese Medicine. Ethical approval was obtained on June 25, 2023. The results were to be published after the conclusion of the trial.

## Methods

### Study Design

**Figure 1:**
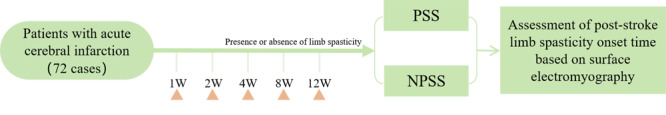
Trial flowchart. Triangles represent the root mean square evaluation of surface electromyography, Modified Ashworth Scale score evaluation, and simplified Fugl-Meyer Assessment Scale score evaluation. NPSS: non-poststroke spasticity; PSS: poststroke spasticity.

The research methodology used in this study was an NCC with a specific emphasis on PSS. The research cohort consisted of approximately 72 patients admitted to the Hunan Integrated Traditional Chinese and Western Medicine Hospital (Hunan University of Chinese Medicine Affiliated Hospital) due to cerebral infarction. A standardized case survey form was designed to gather general information, clinical data about cerebral infarction, and imaging data for both patient groups. Relevant scale assessments were conducted within 24 hours of admission. The root mean square (RMS) values of sEMG, Modified Ashworth Scale (MAS), and Fugl-Meyer Assessment Scale (S-FM) were assessed on the bilateral biceps brachii, abductor pollicis brevis, tibialis anterior, and gastrocnemius muscles of patients at 1, 2, 4, 8, and 12 weeks in both active and passive flexion states upon inclusion. The patients who developed limb spasticity during the 12-week follow-up period were categorized as the spasticity group, while those who did not were the control group. The study flowchart is illustrated in Figure 1. The SPIRIT (Standard Protocol Items: Recommendations for Interventional Trials) schema is depicted in Table 1 (checklist provided in Multimedia Appendix 1).

**Table 1:** Study visit schema.

Research content	Screening period (D_-5_-D_0_^a^)	Observation period (1 week)	Observation period (2 weeks)	Follow-up period (4 weeks)	Follow-up period (8 weeks)	Follow-up period (12 weeks)
Signing an informed consent form	✓					
Reviewing inclusion and exclusion criteria	✓					
Demographic data	✓					
Collecting medical history	✓					
Internal medicine assessment (vital signs)	✓	✓	✓	✓	✓	✓
12-lead electrocardiogram	✓					
MAS^b^ score evaluation		✓	✓	✓	✓	✓
RMS^c^ evaluation of surface electromyography		✓	✓	✓	✓	✓
S-FM^d^ score evaluation		✓	✓	✓	✓	✓

^a^D_–5_-D_0_: day –5 to day 0.

^b^MAS: modified Ashworth scale.

^c^RMS: root mean square.

^d^S-FM: simplified Fugl-Meyer.

### Steps for sEMG

#### Materials and Equipment

**Figure 2:**
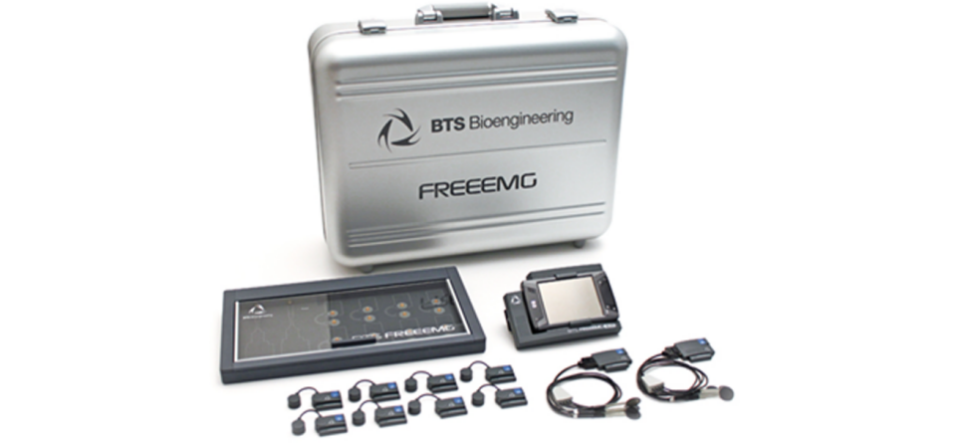
Surface electromyography device.

The sEMG collection and analysis system used in this study was manufactured by BTS (model FreeEMG 300, serial number: 2106-F7 60 0E; Figure 2).

#### Collection Sites

sEMG signals from the biceps brachii, flexor pollicis brevis, and tibialis anterior and gastrocnemius muscles during different states of elbow flexion, thumb flexion, index finger abduction, and ankle dorsiflexion, and plantarflexion were collected [10,11]. EMG waveforms were displayed on the screen in real time during the collection process, allowing the experimenter to verify signal quality and record data.

#### Collection Method

##### Overview

1. Patient preparation: The patient was held lying or sitting for 30 minutes. The strenuous activity was prohibited to prevent muscle fatigue, potentially impacting the experimental results.
2. Skin preparation: The measured area was wiped with a 75% alcohol swab to remove surface oils, increase conductivity, and minimize data error.
3. Electrode configuration: Ag/AgCl surface electrodes (10 mm diameter, 20 mm interelectrode distance) were used and positioned according to the Surface ElectroMyoGraphy for the Non-Invasive Assessment of Muscles (SENIAM) guidelines. Channel A (left side) and Channel B (right side) corresponded to the target muscles: biceps brachii, flexor pollicis brevis, tibialis anterior, and gastrocnemius. The active electrode was placed at the point of maximal muscle belly thickness, with an interelectrode spacing of approximately 1.5-2 cm.
4. Steps: The participant was settled in a sitting or supine position for optimal comfort. The participant was directed to relax completely, ensuring no EMG signals were observed on the oscilloscope. During active and passive muscle contractions, signs were collected from the biceps brachii, flexor pollicis brevis, tibialis anterior, and gastrocnemius muscles. The patient was instructed to perform the movements with the utmost effort and complete them uniformly within 2 seconds. After the signal stabilized, the position was maintained for 3-5 seconds before the movement was repeated. A 25-second cycle was used to collect EMG signals from the muscles on both sides in active and passive states. The participant was permitted to rest after completing 1 cycle. The collection process was repeated 3 times, using the average of the 3 measurements as the final indicator.

##### Biceps Brachii Collection

The examiner fixed the participant’s elbow joint using his left hand and instructed the participant to perform elbow flexion, ensuring the movement was completed uniformly within 2 seconds. Once the signal stabilized, it was maintained for 3-5 seconds and the operation was repeated. EMG signals from both biceps brachii in an active state were collected using a 25-second cycle. The participant’s biceps brachii were fully relaxed. The examiner fixed the participant’s elbow joint using his left hand and instructed him to refrain from applying force. The examiner then used his right hand to move the participant’s elbow, adjusting resistance as necessary to ensure the motion was completed uniformly within 2 seconds. Once the signal stabilized, it was maintained for 3-5 seconds before the action was repeated. A 25-second cycle was implemented to collect EMG signals from both biceps brachii in a passive state. Following 1 cycle, the participant was allowed to rest and the collection was repeated 3 times, using the average of the 3 measurements as the final indicator.

##### Flexor Pollicis Brevis Collection

The examiner fixed the ventral side of the participant’s thumb distal segment with his left hand and instructed the participant to perform thumb flexion, ensuring the movement was completed uniformly within 2 seconds. After the signal stabilized, it was maintained for about 3-5 seconds and the action was repeated. The EMG signals from both flexor pollicis brevis muscles in an active state were collected using a 25-second cycle. The participant’s flexor pollicis brevis muscles were completely relaxed. The examiner fixed the ventral side of the participant’s thumb distal segment using his left hand and instructed the participant not to exert force. The examiner then moved the participant’s thumb using his right hand, adjusting the resistance as necessary to complete the motion uniformly within 2 seconds. After the signal stabilized, it was maintained for 3-5 seconds and the action was repeated. A 25-second cycle collected EMG signals from both flexor pollicis brevis muscles in a passive state. After 1 cycle, the participant was allowed to rest and the collection was repeated 3 times, using the average of the 3 measurements as the final indicator.

##### Tibialis Anterior Collection

The examiner fixed the participant’s tibialis anterior using his left hand and instructed the participant to perform dorsiflexion and plantarflexion, ensuring that movement was completed uniformly within 2 seconds. After the signal stabilized, it was maintained for about 3-5 seconds and the action was repeated. The EMG signals from both tibialis anterior muscles in an active state were collected using a 25-second cycle. The participant’s tibialis anterior muscles were completely relaxed. The examiner fixed the participant’s tibialis anterior with his left hand and instructed him not to apply force. The examiner then moved the participant’s foot with his right hand, adjusting the resistance as necessary to complete the motion uniformly within 2 seconds. After the signal stabilized, it was maintained for 3-5 seconds and the action was repeated. A 25-second cycle collected EMG signals from both tibialis anterior muscles in a passive state. After 1 cycle, the participant was permitted to rest and the collection was repeated 3 times, using the average of the 3 measurements as the final indicator.

##### Gastrocnemius Collection

The examiner fixed the participant’s gastrocnemius using his left hand and instructed the participant to perform dorsiflexion and plantarflexion, ensuring the movement was completed uniformly within 2 seconds. After the signal stabilized, it was maintained for about 3-5 seconds and the action was repeated. The participant’s gastrocnemius muscles were fully relaxed. The examiner fixed the participant’s gastrocnemius using his left hand and counseled him to refrain from exerting force. The examiner then moved the participant’s foot using his right hand, adjusting resistance as needed to complete the motion uniformly within 2 seconds. After the signal stabilized, it was maintained for 3-5 seconds and the action was repeated. The EMG signals were collected from both gastrocnemius muscles in a passive state using a 25-second cycle. Let the participant rest after 1 cycle and the collection was repeated 3 times, using the average of the 3 measurements as the final indicator.

#### Participants and Sample Size

Well-experienced occupational therapists on the team were responsible for appointing potentially eligible patients and individuals interested in this study for further evaluation and consent. If the eligible participants were willing to participate, the project leader (XL) must obtain their signed informed consent.

#### Eligibility Criteria

The eligibility criteria of this study are listed in Textbox 1.

**Eligibility criteria.**

**Inclusion criteria**

- The Western medical diagnosis was consistent with the diagnostic criteria for cerebral infarction (refer to the diagnostic criteria for ischemic stroke in the second edition of the Clinical Management Guidelines for Cerebrovascular Diseases in China, organized by the Chinese Stroke Association in 2023): (1) acute onset; (2) focal neurological deficits, with a few cases showing global neurological deficits; (3) symptoms and signs persist for more than 24 hours; (4) exclusion of nonvascular brain lesions; and (5) brain computed tomography or magnetic resonance imaging excludes hemorrhage and other lesions, with responsible ischemic lesions identified.
- Age>18 years old, regardless of gender.
- Patients who were conscious and had stable vital signs.
- Muscle strength grading was strictly limited to a level of 4 or below.
- The onset of symptoms occurred within 7 days in all patients.
- Patients or their family members needed to sign the informed consent form.

**Exclusion criteria**

- Patients who have been diagnosed with poststroke spasticity.
- Any history of motor dysfunction, such as rheumatoid arthritis, limb surgery, joint deformities, multiple sclerosis, spinal cord injury, or neuromuscular disorders that affect limb movement.
- Inability to comply with the research procedures or follow-up due to mental illness, cognitive impairment, or emotional disorders.
- Women who were pregnant or lactating.
- Severe cognitive impairment caused by noncompliance with treatment.

**Discontinuing criteria**

Withdrawal was a consideration for:

- Those who did not meet the inclusion criteria after enrollment.
- Those who did not attend follow-up visits on time were lost to follow-up, possessed incomplete data, or could not determine treatment efficacy.
- Those who developed severe comorbidities or experienced a recurrent stroke during the trial.

#### Sample Size

This study pre-assumed an effect size relative risk (RR) of approximately 4. By using unmatched case grouping with a case-to-control ratio of 1: 1, assuming a control group exposure rate of 40%, α=.05 (2-sided test), and power (1β)=0.8, the estimated sample size was as follows: the sample sizes for the case group and control group were calculated to be N1=N2=33 using power analysis and sample size software (PASS 15.0; NCSS, LLC). The formula is calculated based on the sample size of the unmatched case-control study:



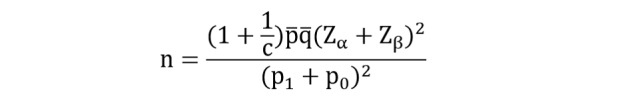



Among them, 
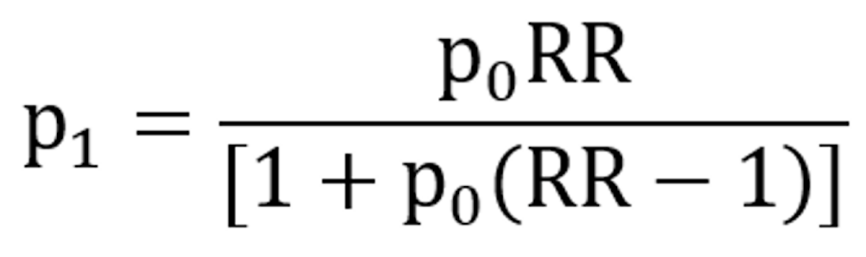
 , 
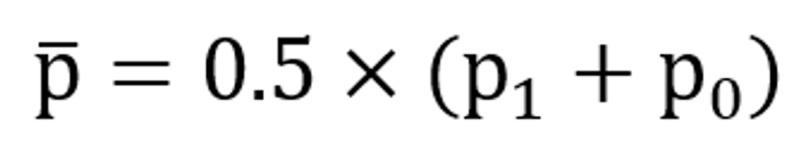
, 
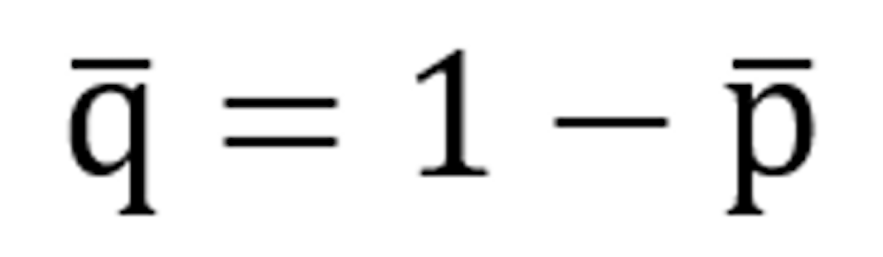
, and c=1 (the control number: the number of cases).

We inflated the total sample size to 72 after considering a nonresponse rate of 10% among the study participants.

#### Unmatched Case Grouping

Patients were selected that met the inclusion criteria were selected. Patients who experienced spasticity events within 12 weeks of follow-up were assigned to the spasticity group. Patients who did not develop spasticity within 12 weeks of follow-up were assigned to the control group based on the spasticity group. Those who did not meet the criteria were excluded.

### Outcomes

#### Primary Outcome: MAS

The MAS is user-friendly, requires no additional tools, and is highly reliable and valid. It is currently the most frequently used tool for assessing muscle tone in research and clinical settings [12]. The MAS grading results were categorized into 6 levels ranging from mild to severe: 0, 1, 1+, 2, 3, and 4. These were quantified as 0, 1, 2, 3, 4, and 5 points, respectively. It is primarily used to evaluate muscle spasticity. The lower the score, the closer it is to normal. If the score is higher, it indicates higher muscle tone and more severe spasticity. Currently, there is no consensus regarding the diagnostic criteria for PSS. Many international studies use the criterion of a modified MAS score of ≥1 to diagnose the PSS [13]. The primary outcome of this study was assessing limb spasticity using MAS within 3 months (Multimedia Appendices 2 and 3).

#### Secondary Outcomes

Secondary outcomes included the evaluation of limb spasticity using the RMS of sEMG and the assessment of limb motor function using the S-FM.

##### RMS

sEMG can reflect the physiological information of muscles in real time, rendering it a powerful tool for rehabilitation assessment, training plan formulation, and monitoring rehabilitation effects. The RMS is the most commonly used time-domain indicator. It is the square root of the mean of the squares of the instantaneous EMG amplitude over a specific period. The larger the RMS value, the greater the muscle strength or tension, which increases its physical significance. Therefore, this research selected RMS as the research indicator.

##### S-FM

The S-FM scale can provide an accurate quantitative assessment of limb function in patients with hemiplegic stroke, with a comprehensive evaluation that includes upper and lower limb movements. There are 6 assessment items for each limb section. Each item is assigned a score of 2 points for full completion, 1 for partial completion, and 0 for no completion. The total score for upper limb movement is 12 points, and the total score for lower limb movement is 12 points, making a combined total of 24 points. The evaluator can evaluate the severity of motor impairment based on the participant’s final score.

Demographic information was collected. These included (1) demographic characteristics, (2) medical history, (3) unhealthful lifestyle history, (4) stroke history, (5) lesion location, and (6) lesion size (Multimedia Appendix 4).

##### Statistical Analysis

The variables from the case investigation form were organized in Microsoft Excel and subsequently imported into SPSS software (version 26.0; IBM Corp) for further statistical analysis. For measurement data that adhered to a normal distribution, the results are expressed as mean and SD, and an independent sample *t* test was used to compare the groups. Measurement data that did not follow a normal distribution are expressed as median (minimum to maximum), and the rank-sum test was implemented for group comparisons. Count data are expressed as numbers (percentages). The rank-sum test was used to compare ordered categorical variables, while unordered categorical variables were compared using the Pearson chi-square test. A *P* value of <.05 indicated statistical significance. The significant data variables identified through the correlation, as mentioned in earlier analyses, were then assigned values appropriate for data processing and subjected to multivariate logistic regression analysis to explore the independent risk factors for spastic paralysis following cerebral infarction (with α=.05 as the test standard).

### Data Collection and Storage

Subsequently, the significant data variables identified through the correlation as mentioned above analyses were assigned values that were appropriate for data processing and subjected to multivariate logistic regression analysis to investigate the independent risk factors for spastic paralysis following cerebral infarction (with α=.05 as the test standard). These paper data were transferred to Microsoft Excel 2020 and EpiData 3.1 software (IBM Corp) for proper management. A total of 2 independent researchers were responsible for double data entry and verification to ensure the data’s accuracy and authenticity. If there were inconsistent data entries or omissions, the original data were rechecked for further verification. After being entered and verified, all data were prohibited from being altered.

Personal data were kept strictly confidential. The participants’ original paper case report forms (CRFs) were securely stored in file cabinets at the research site. Coded identification numbers were used in all reports, data collection, processing, and administrative forms to guarantee participant confidentiality. All paper records with names or personal identification codes were stored separately from documents containing coded numbers. The data were stored on a computer at the research site with a password set by the project leader that was only accessible to the study’s staff. Participants’ personal information was not disclosed without their written consent.

### Trial Status

The trial is in progress and recruiting patients with PSS.

### Quality Control

This study collected all medical records following a standardized CRF to prevent subjective bias. The research process ensured the authenticity, completeness, and consistency of medical record collection. Trained physicians were responsible for collecting all pertinent cases to ensure data accuracy. (1) Patients were informed of the pertinent survey items before data collection. Data collection was initiated only after obtaining the patient’s consent. A professional explanation of terminology was provided to patients during the survey to avoid using suggestive language. (2) The meticulous and precise recording process guaranteed the data’s authenticity. Any required modifications were documented, along with the rationale behind them. (3) All MAS and sEMG assessments in this study were conducted by 2 therapists from our research team, each with over 3 years of experience in neurorehabilitation assessment. (4) Data were entered into an Excel database. Information was promptly recorded, and all indicators were verified to ensure accuracy during each data collection session. (5) Regular validation of research data records was conducted, and the study’s authenticity, safety, and progress were assessed.

### Ethical Considerations

The study was registered in the Chinese Clinical Trials Register (registration number: ChiCTR2300077121) and was approved by the Ethics Committee of the Affiliated Hospital of Hunan Academy of Traditional Chinese Medicine (approval number 202371). Each participant signed a written informed consent form (informed consent is displayed in Multimedia Appendix 5). We strictly adhered to the Declaration of Helsinki. The investigation findings were to be published in a peer-reviewed journal and presented at national or international academic conferences.

## Results

This study was funded by the Chinese Ethnic Medicine Association in October 2023 and is scheduled to conclude in October 2025. Recruitment and enrollment began in November 2023 and are currently ongoing. The study is designed to assess MAS, S-FM, and RMS values at weeks 1, 2, 4, 8, and 12. An increase in MAS and RMS values accompanied by a decrease in S-FM values is considered indicative of the onset of spasticity. Patients who develop limb spasticity are categorized into the spasticity group, while those without spasticity serve as the control group. As of March 2025, a total of 66 participants have been enrolled, with 56 of them having completed the 12-week follow-up assessments of MAS, S-FM, and RMS. We expect to complete patient enrollment and data analysis in December 2025 and aim to publish the results in 2026.

## Discussion

### Anticipated Findings

This clinical study aims to investigate the onset timing of PSS through an NCC design, with a focus on the early identification and prediction of PSS-related factors, ultimately to improve patient prognosis. Stroke is one of the most prevalent causes of long-term disability worldwide [1]. Despite the active rehabilitation treatment, the severity of motor disorders in many patients with stroke continues to vary [14,15]. Limb disharmony is a common symptom of motor dysfunction primarily caused by spasms. This condition impedes the patient’s motor function recovery and increases his economic burden [16]. The data reveal that PSS patients’ medical expenses are increasing 4-fold. Therefore, the onset time of PSS can provide a deeper understanding of its development [17]. sEMG signals can reflect neuromuscular activity to a certain extent due to the varying degrees of correlation between signals and the functional and active states of muscles [18,19]. The sEMG continuously measures the muscle activity in a stationary state and monitors changes in muscle activity during movement [20]. The alterations in sEMG signal characteristics are highly consistent with physiological and pathological changes, which facilitates inference of neuromuscular pathology [21,22] and provides a significant reference value for assessing efficacy in rehabilitation medicine. Studies have demonstrated a significant correlation between the MAS assessments and RMS values in patients with increased upper limb muscle tone post stroke [23]. The assessment of spasticity-related functional impairment largely relies on highly subjective scales. Consequently, we used sEMG to investigate the onset of spasticity within 3 months after a stroke to eliminate errors such as missed diagnoses resulting from subjective assessments.

This study used an NCC design, a hybrid method that integrates features of both traditional case-control and cohort studies. Specifically, it involves conducting a case-control analysis within a previously established cohort under longitudinal observation [24]. The NCC design combines the strengths of cohort and case-control approaches while mitigating the limitations of traditional case-control studies. It offers higher statistical power and testing efficiency, and allows estimation of disease incidence [25]. Compared with conventional case-control studies, NCC studies require a smaller sample size, are easier to conduct, and yield quicker results. Moreover, they enable investigation of multiple etiological factors for a single disease. Since both cases and controls are selected from the same cohort, the comparability between groups is improved, reducing selection bias. In addition, exposure data are collected before disease onset, ensuring temporal causality and minimizing recall bias [26]. Therefore, this study adopted an NCC design to investigate the timing and risk factors of poststroke spasticity.

Currently, there is no internationally recognized standardized treatment regimen for poststroke spastic hemiplegia due to its unclear pathological mechanism [27]. The prognosis of spastic paralysis following cerebral infarction is related to biological factors, pathological factors, and psychosocial factors [28]. Therefore, the treatment and prevention of spastic paralysis following cerebral infarction require comprehensive consideration of multiple factors, including etiology, clinical manifestations, and the specific circumstances of the patient. This study developed a CRF to collect relevant patient data during admission. The spastic hemiplegia occurrence and prognosis following cerebral infarction are influenced by various factors, including age, gender, etiology, infarct location, scale scores, and early treatment. These factors should be comprehensively considered during treatment to develop individualized treatment plans that are aimed at improving the prognosis of patients with spastic hemiplegia following cerebral infarction. Currently, there is a lack of conclusive evidence regarding the risk factors for patients with spastic hemiplegia, and the impact of these factors on clinical treatment remains uncertain. Future research should investigate the mechanisms of these factors further and explore relevant treatment strategies to direct clinical practice more effectively.

Existing evidence-based studies indicate that poststroke spasticity typically emerges gradually within 1 to 3 months after stroke onset. However, in some cases, it can be observed as early as 1 to 3 weeks, depending on the location and severity of the brain lesion. The incidence increases with disease progression, with approximately 30% to 40% of patients developing spasticity within the first 3 months [29]. Wissel et al conducted a prospective observational study in which a cohort of 103 patients with stroke was followed up at a median of 6 days, 6 weeks, and 16 weeks post stroke. The results showed that 24.5% of patients exhibited increased muscle tone within 2 weeks after stroke, and approximately 19% to 39% developed spasticity within 3 months after onset [30]. Lundström et al conducted a 5-year follow-up study on patients with stroke and found that the incidence of spasticity peaked within the first year poststroke, ranging from 25% to 40%, and then stabilized thereafter [31].

According to the 2018 “China Stroke Prevention and Treatment Report,” the geographical distribution of stroke in China shows a pattern of “higher in the north, lower in the south, and prominent in the central regions.” From 1990 to 2017, the number of stroke-related deaths in Hunan province increased annually, and the overall disease burden of stroke in the province has shown an upward trend. The monitoring data from Hunan’s cerebrovascular disease population indicated that the incidence and prevalence of transient ischemic attack in Hunan are higher than the national average. However, as the study sample was exclusively drawn from the Hunan Provincial Hospital of Integrated Traditional Chinese and Western Medicine, the homogeneity of the sample limits the ability to perform regional comparative analyses. To enhance the generalizability of our findings, we have initiated the establishment of a multicenter clinical research network. Letters of intent have been signed with several tertiary hospitals across different regions, and the second phase of data collection is being planned accordingly.

### Conclusion

This nested case-control study investigates the onset timing and predictive factors of PSS within the critical 3-month window using objective sEMG assessment. If key predictive factors and the early onset window are identified, this approach could provide an objective basis for timely clinical intervention in PSS, ultimately improving functional recovery and reducing the significant associated health care burden.

## Appendix

Multimedia Appendix 1 SPIRIT (Standard Protocol Items: Recommendations for Interventional Trials) checklist.

Multimedia Appendix 2 Muscle strength measurement.

Multimedia Appendix 3 Modified Ashworth Scale.

Multimedia Appendix 4 Simplified Fugl-Meyer Scale.

Multimedia Appendix 5 Clinical information collection form.

## Abbreviations

CRF: case report form
MAS: Modified Ashworth Scale
NCC: nested case-control
PSS: poststroke spasticity
RMS: root mean square
RR: relative risk
sEMG: surface electromyography
S-FM: simplified Fugl-Meyer
SENIAM: Surface ElectroMyoGraphy for the Non-Invasive Assessment of Muscles
SPIRIT: Standard Protocol Items: Recommendations for Interventional Trials

## Data Availability

The datasets generated or analyzed during this study are available from the corresponding author on reasonable request.
