# Supplementary material for: Exploring the Time to Onset and Early Predictors of Poststroke Spasticity Combined With Surface Electromyography: Protocol for a Nested Case-Control Study
Source: JMIR Res Protoc. 2025 Aug 5;14:e65829. doi: 10.2196/65829 (PMC12365559; doi:10.2196/65829)
Supplement: Multimedia Appendix 2 [file resprot_v14i1e65829_app2.docx]

| Muscle strength | |
| --- | --- |
| Level 0，No visible or palpable muscle contractions；  Level 1，Palpable muscles contract slightly, but there is no joint movement；  Level 2，In a gravity-free position, you can do exercises with a full range of motion；  Level 3，Able to resist gravity to do exercises with full range of motion of the joints, but not resistance；  Level 4，Able to resist gravity and certain resistance movement；  Level 5，Movement that can resist gravity and sufficient resistance。 | 口 Level 0  口 Level 1  口 Level 2  口 Level 3  口 Level 4  口 Level 5 |
