# Supplementary material for: Exploring the Time to Onset and Early Predictors of Poststroke Spasticity Combined With Surface Electromyography: Protocol for a Nested Case-Control Study
Source: JMIR Res Protoc. 2025 Aug 5;14:e65829. doi: 10.2196/65829 (PMC12365559; doi:10.2196/65829)
Supplement: Multimedia Appendix 3 [file resprot_v14i1e65829_app3.docx]

| Level | Muscle tone | standard |
| --- | --- | --- |
| 0 | Muscle tone does not increase | Passive movement of the affected limb is performed without resistance over the entire range |
| 1 | Muscle tone increases slightly | There is slight resistance when passively moving the affected limb to the terminal end |
| 1+ | Muscle tone increases slightly | There is a slight "stuck" sensation in the anterior 1/2 range of motion (ROM) and slight resistance in the posterior 1/2 ROM when the affected limb is passively moved |
| 2 | Slight increase in muscle tone | Passively moving the affected limb has resistance in most of the ROM, but it can still move |
| 3 | Moderate increase in muscle tone | Passive movement of the affected limb has resistance throughout the ROM, making movement difficult |
| 4 | Muscle tone is highly increased | The affected limb is stiff and resistant, making passive movement difficult |
| Rating | \|_\|Level | |
