# Supplementary material for: Exploring the Time to Onset and Early Predictors of Poststroke Spasticity Combined With Surface Electromyography: Protocol for a Nested Case-Control Study
Source: JMIR Res Protoc. 2025 Aug 5;14:e65829. doi: 10.2196/65829 (PMC12365559; doi:10.2196/65829)
Supplement: Multimedia Appendix 4 [file resprot_v14i1e65829_app4.docx]

| **Simplified Fugl-Meyer** | | | | | | | |
| --- | --- | --- | --- | --- | --- | --- | --- |
| **Assessment items** | **0 points** | | **1 points** | | | **2 points** | **Score** |
| **Upper limbs (seated position)** | | | | | | | |
| Shoulder Elevation | **Unable to perform** | | **Partially completed** | | | **Fully completed without interruption** |  |
| Shoulder flexion 90-180 degrees (elbow in extension, forearm neutral) | Initial elbow flexion or shoulder abduction | | In shoulder flexion, elbow flexion and shoulder abduction | | | **Successfully completed** |  |
| Elbow extension | **Unable to perform** | | **Partially completed** | | | **Fully completed without interruption** |  |
| Forearm supination, supination (Shoulder 0 degrees, elbow 90 degrees) | The elbow cannot be flexed or the forearm cannot be supinated | | The shoulder and elbow positions are correct, and they can basically pronate and supinate | | | **Successfully completed** |  |
| Wrist flexion and extension (at 90 degrees of elbow flexion and 0 degrees of shoulder flexion) | Voluntary movement is not possible | | Patients are unable to actively move the wrist joint across the joint | | | The subject is able to progress smoothly and without pause |  |
| Lateral pinching (thumb adduction with all joints straightened) | Unable to perform | | Can pinch a piece of paper with your thumb and forefinger, but cannot resist the pull | | | Grip the paper firmly |  |
| Scores of the upper limbs | |  | | | | | |
| **lower limbs** | | | | | | | |
| Hip flexion (supine position) | | Unable to perform | | Partial movement | Fully carried out | |  |
| Knee flexion (sitting) | | No active movement | | The knee can be flexed from a slightly extended position but not more than 90 degrees | Knee flexion > 90 degrees | |  |
| Knee flexion (upright) | | The knee cannot be flexed in the hip extension position | | The hip is not flexed, the knee can be flexed but less than 90 degrees, or the hip is flexed during progression | Able to move freely | |  |
| Ankle dorsiflexion (sitting) | | Unable to actively move | | Able to partially dorsiflexion | Able to fully dorsiflexion | |  |
| Ankle plantar flexion (supine position) | | There is no movement | | Weak movements | Almost identical to the contralateral side | |  |
| Heel and tibial test speed (5 consecutive repetitions) | | 6s slower than the healthy side | | 2-5s slower than the healthy side | The difference between the two sides is less than 2s | |  |
| Score of the lower limbs | |  | | | | | |
| **Total scores** | |  | | | | | |
