# Supplementary material for: Exploring the Time to Onset and Early Predictors of Poststroke Spasticity Combined With Surface Electromyography: Protocol for a Nested Case-Control Study
Source: JMIR Res Protoc. 2025 Aug 5;14:e65829. doi: 10.2196/65829 (PMC12365559; doi:10.2196/65829)
Supplement: Multimedia Appendix 5 [file resprot_v14i1e65829_app5.docx]

| **Informed consent signing** |
| --- |
| **Has the patient signed the patient informed consent form？**  □Yes □No Time：\|_\|_\|_\|_\|/\|_\|_\|/\|_\|_\| (Year/Month/Day) |
| **General information**  Gender：□Male □Female Date of birth：\|_\|_\|_\|_\|/\|_\|_\|/\|_\|_\| (Year/Month/Day)  Height：\|_\|_\|_\|cm Weight：\|_\|_\|_\|.\|_\|kg |
| Vital signs  Temperature \|_\|_\|.\|_\|℃ Heart rate \|_\|_\|_\|times/min  Breathe \|_\|_\|times/min Systolic/diastolic blood pressure \|_\|_\|_\|/\|_\|_\|_\|mmHg |

| **Past medical history** | **Yes No** |
| --- | --- |
| Hypertension | □ □ |
| Diabetes | □ □ |
| Hyperlipidemia | □ □ |
| Coronary atherosclerotic heart disease | □ □ |
| **Personal life history** | **Yes No** |
| History of smoking | □ □ |
| Quit smoking | □ □ |
| History of alcohol consumption | □ □ |
| On the wagon | □ □ |

| **Clinical data related to cerebral infarction** | |
| --- | --- |
| **History of stroke** | **Yes No** |
| Whether you had a first stroke | □ □ |

| **Imaging data** | |
| --- | --- |
| Site of injury in cerebral infarction | 口Basal ganglia  口Frontotemporoparietal lobe  口 Lobes  口 Thalamus  口 Brain stem  口 Cerebellum  口 midbrain |
| Whether it is a massive cerebral infarction | 口 Yes 口No |
